# The risk of pedestrian collisions with peripheral visual field loss

**DOI:** 10.1167/16.15.5

**Published:** 2016-12-05

**Authors:** Eli Peli, Henry Apfelbaum, Eliot L. Berson, Robert B. Goldstein

**Affiliations:** eli_peli@meei.harvard.edu http://serinet.meei.harvard.edu/faculty/peli/; Henry_Apfelbaum@meei.harvard.edu; Eliot_Berson1@meei.harvard.edu; Robert_Goldstein@meei.harvard.edu; Schepens Eye Research Institute, Massachusetts Eye and Ear, Harvard Medical School, Boston, MA, USA; Schepens Eye Research Institute, Massachusetts Eye and Ear, Harvard Medical School, Boston, MA, USA; Massachusetts Eye and Ear, Harvard Medical School, Boston, MA, USA; Schepens Eye Research Institute, Massachusetts Eye and Ear, Harvard Medical School, Boston, MA, USA

**Keywords:** *low vision*, *rehabilitation*, *peripheral field loss*, *retinitis pigmentosa*, *residual vision*, *modeling collision*

## Abstract

Patients with peripheral field loss complain of colliding with other pedestrians in open-space environments such as shopping malls. Field expansion devices (e.g., prisms) can create artificial peripheral islands of vision. We investigated the visual angle at which these islands can be most effective for avoiding pedestrian collisions, by modeling the collision risk density as a function of bearing angle of pedestrians relative to the patient. Pedestrians at all possible locations were assumed to be moving in all directions with equal probability within a reasonable range of walking speeds. The risk density was found to be highly anisotropic. It peaked at ≈45° eccentricity. Increasing pedestrian speed range shifted the risk to higher eccentricities. The risk density is independent of time to collision. The model results were compared to the binocular residual peripheral island locations of 42 patients with forms of retinitis pigmentosa. The natural residual island prevalence also peaked nasally at about 45° but temporally at about 75°. This asymmetry resulted in a complementary coverage of the binocular field of view. Natural residual binocular island eccentricities seem well matched to the collision-risk density function, optimizing detection of other walking pedestrians (nasally) and of faster hazards (temporally). Field expansion prism devices will be most effective if they can create artificial peripheral islands at about 45° eccentricities. The collision risk and residual island findings raise interesting questions about normal visual development.

## Introduction

Patients with retinitis pigmentosa (RP) and similar diseases, such as Usher syndrome, choroideremia, and advanced glaucoma, typically maintain good central visual acuity but lose peripheral visual field. Peripheral field loss (PFL) results in difficulties with orientation and mobility (Geruschat & Turano, 2002; Turano, Geruschat, Stahl, & Massof, 1999). Mobility difficulties of people with PFL include tripping over stationary obstacles in their path and collisions with other pedestrians (Haymes, Johnston, & Heyes, 2002; Lisboa et al., 2013; Lovie-Kitchin, Mainstone, Robinson, & Brown, 1990; Lovie-Kitchin, Soong, Hassan, & Woods, 2010; Turano et al., 1999). Many patients with PFL also have impaired night vision. The analyses reported in this article are pursuant to our efforts to improve mobility safety (in daylight/photopic conditions) for patients with PFL through the use of prisms in spectacles (Peli & Jung, 2016; Woods, Giorgi, Berson, & Peli, 2010). The prisms will expand the field of view to improve hazard detection, primarily when walking (see Peli & Jung, 2016, for explanation of how the prism spectacles create artificial islands of view into the blind periphery). Here, we focus on the question of where those views would be most effective.

The effects of PFL are often first noticed when residual field diameter shrinks below 40°, while the long cane generally is not adopted before legal blindness is declared and the residual field has dropped below 20°. We expect our prisms to be effective for patients with residual central field diameters between 10° and 30°. Assuming a typical residual field area loss of a few percent per year (Grover, Fishman, Anderson, Alexander, & Derlacki, 1997), patients might benefit for decades.

The long cane addresses tripping difficulties, but is limited in the amount of warning it can provide for pedestrian collisions, which should be averted before contact with the cane. While the cane itself marks the user as blind or visually impaired, alerting other pedestrians to avoid the collisions, many legally-blind people are reluctant to use the cane, precisely because of this marking effect (Sacks, 2010), and thus approaching pedestrians are provided no indication that they may not be seen.

We do not expect prisms to help patients with tripping hazards, as even very high-power prisms (base down) cannot provide the hazard view needed, as shown in the Results section. Without the cane, prisms could provide a view farther ahead for detecting an impending tripping hazard, but the residual central island of vision is likely already sufficient for detection at farther distances where the residual field includes a larger span along the path. It has been shown that patients scan downward when they walk in an environment that may include tripping hazards (e.g., on sidewalks) but not when walking in an environment where tripping hazards are unlikely (e.g., inside a large office building; Luo, Vargas-Martin, & Peli, 2008; Vargas-Martin & Peli, 2006).

Oncoming potentially colliding pedestrians are similarly detectable from a distance if they are walking on a path almost parallel to that of the patient. Paths constrained to parallelism are common on sidewalks or walkways and corridors. Patients with visual field loss mention collisions with other people approaching from the side (Geruschat & Turano, 2002). Anecdotally, this occurs in wider and crowded school corridors and supermarket aisles, but greater concern is cited in many open space environments, particularly in shopping malls and bus, train, and airport terminals, as well as city plazas and parks. In those environments pedestrian movements are less regulated and pedestrians may approach the patient from any direction. We are not aware of any study questionnaires that make these distinctions, but results from studies on mobility courses seem consistent with that observation (Haymes, Guest, Heyes, & Johnston, 1996; Kuyk, Elliott, & Fuhr, 1998a, 1998b; Lovie-Kitchin et al., 1990). Helping patients with prismatic solutions in these unconstrained environments is thus our focus, and this article analyzes how best to do that within the constraints of severely limited residual central fields and available prism properties.

Our studies and others had shown that PFL patients do not compensate for their loss by using wider lateral eye scans than normally sighted people (even though they see much less with each scan) and spend most of the time at primary gaze (Iorizzo, Riley, Hayhoe, & Huxlin, 2011; Luo & Peli, 2006; Vargas-Martin & Peli, 2006). We did not find reliable studies demonstrating that training patients to use wider scans transfers specifically to natural mobility conditions. Therefore, identifying the direction of highest risk and providing islands of vision in that direction when the patient is gazing straight ahead, as is most common during walking (Vargas-Martin & Peli, 2006), may be the optimal strategy. That is the challenge addressed in this article.

While prisms may be helpful when searching for static objects, we are primarily hoping for them to improve the safety of patients walking in the dynamic open space environments where pedestrians may approach from any direction. We model the walking directions of pedestrians in all locations who would likely collide with the patient. Our model benefits from the observation that if patient and pedestrian are proceeding on straight paths, each at a constant speed, a pedestrian on a collision course with the patient will remain at a constant bearing angle relative to the patient's heading (Regan & Kaushal, 1994). The model we present below determines the bearings from the patient of the highest pedestrian collision threats. Directing our prism-extended views toward those bearings would optimize pedestrian hazard detection.

It has long been known that the peripheral visual field does not simply shrink uniformly and centripetally as the disease progresses. Many patients go through a period with one or more peripheral residual islands of vision remaining, disconnected from the residual central field. Do those islands provide hazard detection? If so, where are they located, in relation to the collision risk derived from our model, and, for patients who do not have those islands, can we target our artificial islands there? Despite countless thousands of visual fields that have been measured for diagnostic and prognostic counseling, little has been published about the location and functionality of those natural peripheral islands. Fishman, Grover, and colleagues published several studies identifying patterns of PFL progression in RP patients (Fishman, Bozbeyoglu, Massof, & Kimberling, 2007; Grover et al., 1997; Grover, Fishman, & Brown, 1998). The most common pattern they identified starts as a midperiphery complete or incomplete ring scotoma, which over time grows inwards and outwards, eventually fragmenting and consuming any remaining islands of peripheral vision, until only the central field remains. However, they did not try to identify the functionality of the islands, nor was there a quantified analysis of the island sizes and locations. Importantly, only the monocular fields were considered in these papers and the binocular fields were not even mentioned. In one article where the binocular field was considered (Fishman, Anderson, Stinson, & Haque, 1981) the binocular “total horizontal visual field diameter” was calculated as the sum of the horizontal residual visual field of both eyes. A similar summation of both eyes, horizontal field extent was applied for visual fields in other studies of RP (Szlyk, Fishman, Master, & Alexander, 1991) and glaucoma (Szlyk, Taglia, Paliga, Edwards, & Wilensky, 2002). Such summation is correct only if the residual fields of both eyes are completely nonoverlapping. Yet, binocular fields were calculated, and used properly in one study of driving by patients with RP (Szlyk, Alexander, Severing, & Fishman, 1992).

Others have analyzed the impact of field loss on mobility (Hassan, Hicks, Lei, & Turano, 2007; Kuyk & Elliott, 1999; Kuyk et al., 1998a; Leat & Lovie-Kitchin, 2006; Turano et al., 1999) or activities of daily living (Haymes et al., 2002; Lisboa et al., 2013; Lovie-Kitchin et al., 2010; Yanagisawa, Kato, Kunimatsu, Tamura, & Ochiai, 2011), but almost always reduced the complex perimetry results to a single scalar value related to field area, and thus obscured any specific information about the role of islands, per se. Haymes et al. (1996) did analyze fields in terms of increasing rings of peripheral eccentricity, but the contribution of just the island regions could not be isolated. This was a rare study in which actual pedestrian encounters were possible, but the contribution of peripheral residual islands to the results cannot be determined.

Lovie-Kitchin et al. (1990) identified visual field areas important for mobility. They divided the visual field into 15 thoughtfully delineated equal areas and were able to report correlations between the separate areas with percentage of preferred walking speed and numbers of errors in their indoor obstacle course. Their results are consistent with our general hypothesis that the region that some islands of vision occupy are important for mobility, but the area boundaries they used blur close comparison, and their mobility course did not include dynamic hazards such as walking pedestrians. Over 50 publications have cited this article, but none provided further insights into peripheral island functionality.

Prior literature left our question of natural island locations and utility unanswered. To that end, we report here on the peripheral fields of a convenience sample of patients with residual islands. We compare island location and extent with the regions of greatest pedestrian hazard predicted by our collision risk model.

## Methods

### Collision risk model

The collision risk model starts with calculating the point risk posed by any pedestrian in the open space environment. We first describe the geometric conditions that apply if a pedestrian is on course to collide with the patient. Then we introduce a *wedge diagram*, which shows the range of walking directions by all pedestrians starting at a given location that will lead to a collision. The angular range is determined by location, range of walking speeds, and maximum time to collision considered. That angular range, as a fraction of the semicircle of all pedestrian headings toward the patient's path, represents the *point risk* of collisions from that location. As explained below, we eventually derive a *risk density function* as a function of the pedestrian bearing angle at the patient position. The area under risk density curve between two bearings represents the percentage of total risk that a window of vision between those eccentricities would be able to monitor.

### Collision geometry

**Figure 1:**
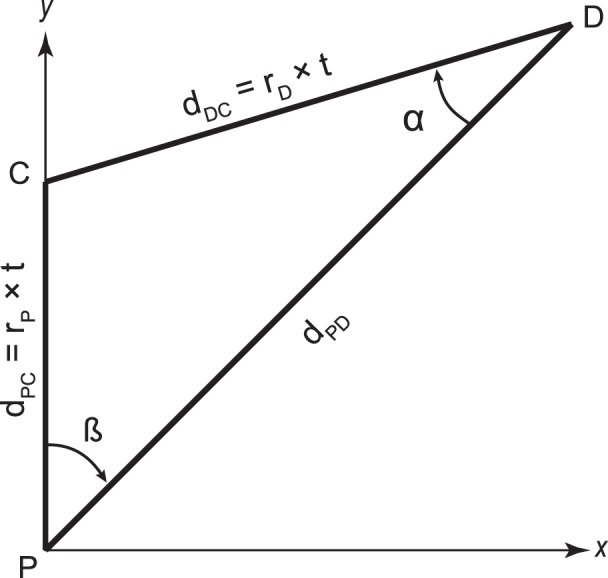
A Patient, P, walks in the *y* direction toward C for *t* seconds. A peDestrian, D, also walks toward C at a speed that will cause him to collide with the patient at C. Under the simplifying assumptions that both maintain constant speed and direction, D remains at a constant bearing angle *β* with respect to P as they approach collision.

Figure 1 shows a patient, P, at an arbitrary location (0, 0) and moving in the *y* direction at a constant speed, r_P_. Pedestrians in any location around the patient may be walking in any direction and at various speeds. Some are on a collision course with the patient. A pedestrian, D, located at a distance d_PD_ and bearing angle *β* from the patient, walking at a constant speed, r_D_*,* toward point C, is on a collision course if the patient and pedestrian arrive at C at the same time. Assuming that we know the relative ratio of the pedestrian and patient speeds, r_D_/r_P,_ the triangle P-C-D is fully determined. All angles, distances, and times relating to the collision are calculable. Note that with constant speeds and directions, the triangle shrinks as patient and pedestrian approach collision point C, but remains similar (in the Euclidian sense) throughout. Thus, the pedestrian remains at a constant bearing with respect to the patient (Regan & Gray, 2000), so for a patient gazing steadily along the path, the pedestrian remains at a constant eccentricity in the patient's field of view. The pedestrian also looms larger, but we ignore the finite size of the patient and pedestrians in the simplifications made for this model.

### Wedge diagrams: Point risk

**Figure 2:**
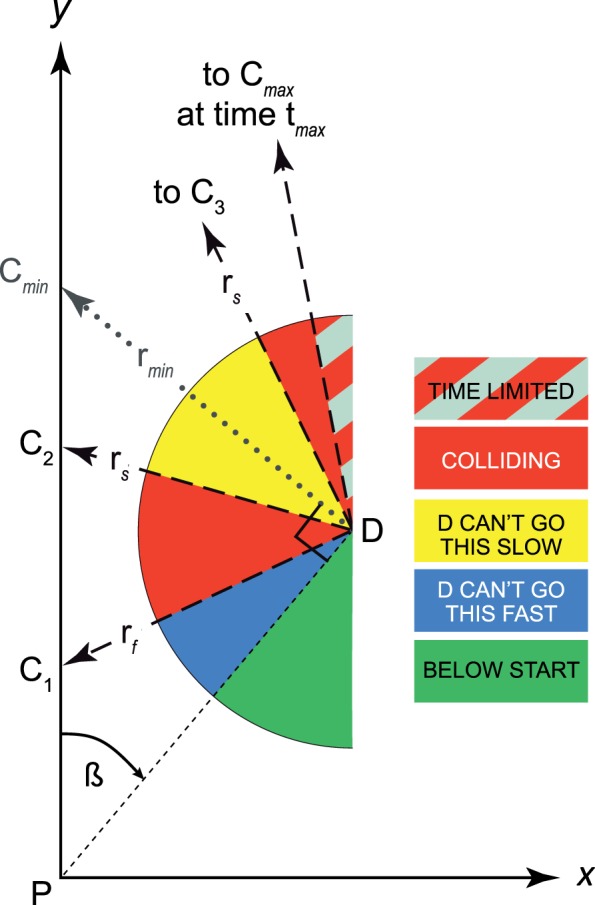
The wedge diagram identifies (in red) the headings a pedestrian, D, starting at a given single location can take to collide with the patient, P, within assumed speed and time constraints. Headings that cannot result in a constrained collision are color coded to indicate the applicable constraint. Collisions in the blue area would require D to walk faster than the high speed limit, r*_f_*, while those in the yellow area would require walking slower that the low speed limit, r*_s_*. The gray striped area represents paths that require longer than the maximum time to collision (t*_max_*), even though the speed constraints are met. The percentage of red area (without gray stripes) thus represents the relative risk of collisions from that location. Pedestrian speed decreases from infinite at *α* = 0 (heading directly at the patient start point), to equal r_P_ when *α* = *β* (not shown), reaching a minimum speed r*_min_* at *α* = 90°, then increases to equal r_P_ again and collide at infinite time and distance on a parallel path. This example uses r_P_ = 1 m/s, r*_f_* = 1.5 m/s, r*_s_* = 0.7 m/s, t*_max_* = 5 s, for pedestrians at *β* = 40° and d_PD_ = 1.3 m, resulting in a point risk of 0.31. The wedge diagram would be the same for any distance along that bearing angle, except for the *α* angle and collision point that t*_max_* intercedes. (The wedges would also be unchanged if the speeds change but the ratios of r*_f_*/r_P_ and r*_s_*/r_P_ do not change.)

By placing some reasonable constraints on the range of the relative speeds of pedestrians (r_D_) to patient (r_P_), and further setting a limit on the time to collision (t*_max_*), we can calculate the range of headings from a given pedestrian location over which collisions (of interest) with the patient are possible. We plot that range in red in the wedge diagram (Figure 2). Collisions can occur under these constraints along the patient path only from C_1_ to C_2_ and from C_3_ to C*_max_* (but for farther pedestrians, C*_max_*, the collision point limited by t*_max_*, may intercede before C_2_ or C_1_). The percentage that the red wedges occupy of the total wedge diagram area (or angular range) thus forms the point risk from that pedestrian location.

To provide a better understanding of the dynamics involved, the wedge diagram of Figure 2 also encodes the reasons other pedestrians heading from that location are excluded from colliding with the patient (pedestrians would have to walk too fast or slow to collide, take too long, or not be headed to cross the patient path). That detail is not shown in the subsequent analyses, which only use the red wedges. Appendix A explains the regions more fully and provides the full derivation of the wedge diagrams and point risk.

The wedge diagram only shows collisions with respect to pedestrians positioned to the right of the patient. For simplicity, we do not show diagrams for the symmetrical situation that occurs to the left, nor do we show the right semicircle of the diagrams (where the pedestrians are necessarily walking away from the patient's path).

### Bearing risk

**Figure 3:**
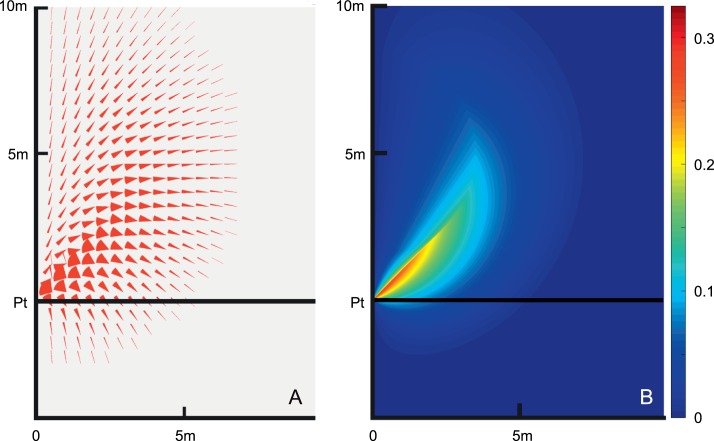
(A) The patient starts at (*x*, *y*) = (0, 0) and walks with increasing *y*. For each pedestrian position in the figure, extending from *x* = 0 to 10 m and *y* = −4 to 10 m, in 0.5-m steps, the angular range of collision hazards of interest is shown in red. In this example, the patient walks at 1 m/s and the pedestrians walk between 0.7 and 1.5 m/s. Collisions that would require times longer than 5 s are not shown. In the region below *y* = 0, the pedestrians start behind the patient, and remain behind the patient to collide. The patient would not be considered responsible for avoiding this type of collision. (B) The point risk (red wedge area) is calculated and shown with color representing the risk. To create this map, the wedge diagram shown in (A) is recalculated at a resolution of 0.02 m.

Figure 3A plots the red wedges of the wedge diagram for each pedestrian location relative to the patient on a grid with half-meter resolution. The patient walks at 1 m/s and the pedestrians can walk between 0.7 and 1.5 m/s. A limit of 5 s is placed on time to collision. (Additional cases are considered in the Results section.) The amount of red in an area provides a measure of relative risk from that area.

Figure 3B provides a risk contour heat map. The wedges have been replaced by a color scale representing the point risk (red wedge area) at each location and a much finer grid resolution is used.

These diagrams illustrate the large variability of collision risk from different directions and distances, and thus give a sense of where pedestrian hazard detection is most needed. We are, however, interested in the risk as a function of pedestrians' bearing angle from the patient, rather than the risk as a function of their two-dimensional spatial location, as the bearing angle represents the location of the colliding pedestrian in the visual field of a patient fixating on the path ahead (which is where most gaze time is spent). For a pie slice between a given bearing angle *β* and *β* + Δ*β*, we calculate the *bearing risk* for the slice by summing the point risks at each polar coordinate point (*ρ*, *β*) multiplied by *ρ*, in steps of 0.02 m, and dividing that by the area of the pie slice. This represents risk in the pie slice. We calculate bearing risk for each *β* between 0° and 90° with Δ*β* increment of 0.1°. The summation at each bearing ends when the risk declines to 0 due to the time constraint.

### Risk density function

**Figure 4:**
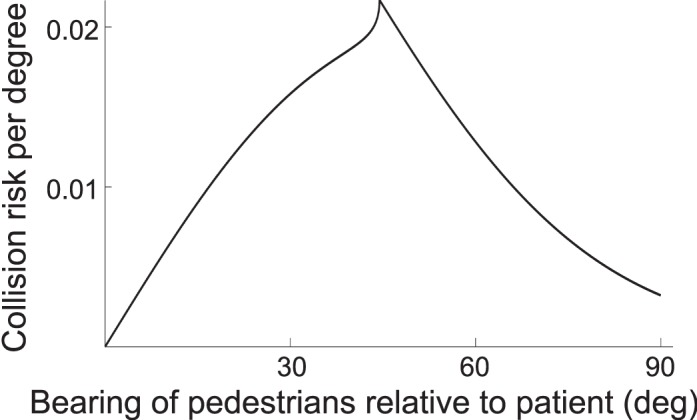
Collision risk as a function of bearing relative to the patient. Total area under the curve is normalized to 1, representing all risks in the quadrant of concern. The percentage area under the curve between any two bearings can thus represent the amount of risk that comes from a window of vision monitoring that range. (Same parameters as in Figure 3.)

To determine percent of risk within a range of bearing angles, we normalize the bearing risks so that the area under the bearing curve is 1, resulting in a risk density as a function of bearing. That is done by summing the bearing risks in the range of 0° to 90°, then dividing each bearing slice by that sum to get the contribution of each slice to total risk. This collision risk density as a function of bearing angle *β* is plotted in Figure 4. The percentage area under the curve between any two bearing angles thus represents the percentage of total risk that a window of visibility in that angle range would monitor for the patient. We don't include the area behind the patient, as it is the pedestrians' responsibility to avoid colliding from behind.

### Natural residual island locations

Patient data were obtained from the records of the Berman-Gund Laboratory of the Massachusetts Eye and Ear Infirmary. The convenience sample was selected from their database using the following criteria: In at least one eye, there was a visual acuity of 20/60 or better, the residual central field area was equivalent to a circular residual field of 20° diameter or smaller, and the total field area was equivalent to a circular diameter of 30° or greater. The latter condition assured that the records selected included residual peripheral islands in at least one eye. With every record, we obtained demographic information and the associated diagnoses, hard copies of the source Goldmann V4e perimetry plots, and separate digital files each with a list of coordinates tracing a boundary (isopter) of the shapes in the residual fields of each eye. The files were coded to indicate whether they bounded a seeing or nonseeing area (a scotoma). When appropriately combined, these outlines identify the shape of each area of a residual island of seeing, including separately marked central fields. The coordinate files had been routinely digitized and preserved from the Goldmann perimetry using the “Image” image processing program developed by the National Institutes of Health and macros developed at Berman-Gund. Initially, 67 patients were identified. Of those 67 patients, 42 were included in our analyses. We excluded patients who did not meet the inclusion criteria in both eyes, because of our interest in binocular field coverage. We excluded a few files that would have required undue effort to include (largely due to digitizing problems caused by manual annotations overlapping the isopters). Several patients had fellow eyes that did not strictly meet the inclusion criteria, such as ones with a connection between the central field and a peripheral island, resulting in a coding of the entire structure as the central field (see Appendix B). Since problems like that had no material effect on our analyses, they were included. For the included 42 subjects, ages 32–89 years (average 52), the median visual acuity was 20/30 (range 20/20 to 20/60). There were 30 patients with various RP diseases (73%), four with choroideremia (10%), and the remaining seven with other miscellaneous diagnoses (17%). The visual fields and other data were de-identified, so no informed consents were required. The Berman-Gund lab did have permission to provide the data for research, and all procedures conformed to the Declaration of Helsinki.

The coordinate files for each patient eye were read into Microsoft Excel and replotted, and Excel macros we created attempted to fill in each shape (within its outer and inner boundaries). Errors trapped in this process revealed problems with the coordinates in some cases, especially if callout annotations touched the traced outlines. In each such case, the coordinates were laboriously edited by hand to eliminate the errors while remaining as true as possible to the original data. We then compared all plots with the original perimetry to ensure that they were correct and complete, and corrected a few miscodings of left eye (LE) and right eye (RE).

Coding in the coordinate file names allowed the Excel program to pair the coordinate file for each seeing region of an eye with any scotomas contained within it. These outlines were plotted with 1° resolution in an array of cells representing −130° to +130° of visual field horizontally and vertically. The program then filled in the cells of the seeing area and counted the cells to quantify the area of that patch of vision. The outlines of the seeing areas of each eye were then combined, essentially reproducing the original Goldmann plot for the eye (Appendix B). Cleaned coordinate data were then exported to MATLAB (MathWorks, Natick, MA) for for further processing to produce the diagrams and statistics reported in Results.

## Results

### Collision risk

**Figure 5:**
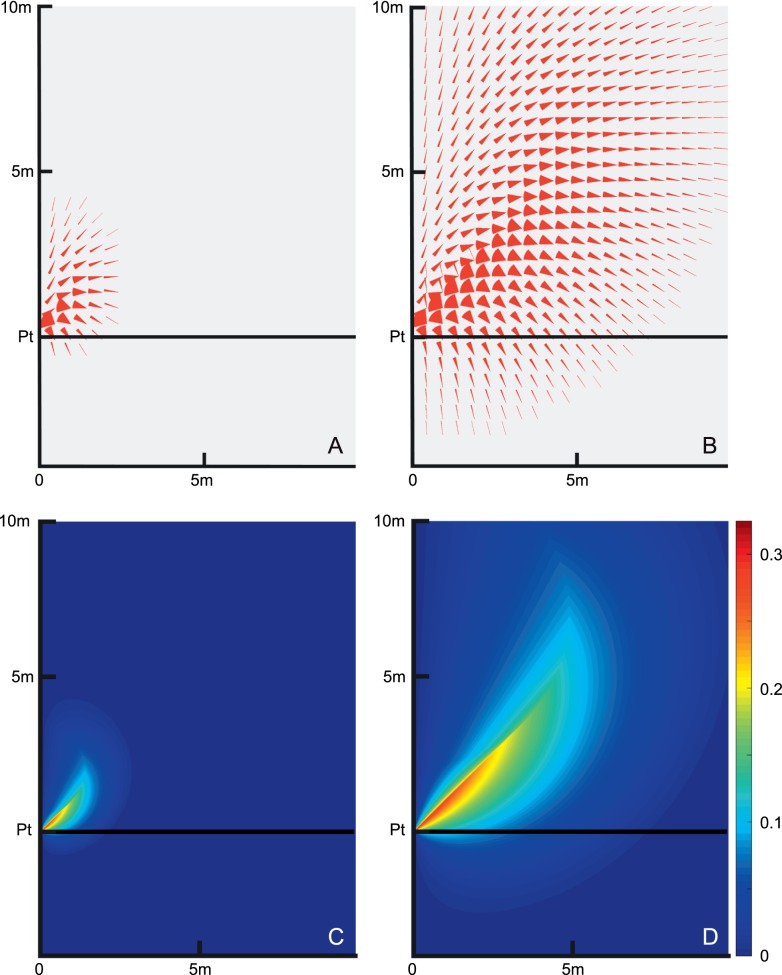
Effect of the maximum time to collision, t*_max_*. (A) With t*_max_* = 2 s, calculated on a 0.5-m grid, the angular range of possible collisions (point risk) is shown in red wedges. (B) Same as Panel (A), but for t*_max_* = 7 s. Collisions are possible with pedestrians that start much farther from the patient. (C) Same as Panel (A), but rendered on a high resolution grid of 0.02 m and the size of the collision wedges is represented by the color coding shown in the scale. (D) Same as Panel (C), but for t*_max_* = 7 s. Despite different t*_max_* values, the risk density as a function of the bearing angle *β* calculated from (C) and (D) is identical to the risk density function shown in Figure 4 for t*_max_* = 5 s.

The collision risk density in Figure 4 illustrates the level of risk associated with walking pedestrians starting from all spatial locations to the right of the walking patient as a function of the bearing angle. As seen in Figure 3, the risk also varies with distance at each bearing, with more risk at some distances and less in others, and the effect varies with bearing. The risk level is highly anisotropic. As might be anticipated, closer pedestrians usually pose higher risk of collision, though that effect is largely a function of t*_max_*, which is the limiting factor for more distant pedestrians. This is illustrated by varying t*_max_* in Figure 5. With a shorter t*_max_* = 2 s, the spatial extent with collision risks and the level of risk at most locations is substantially reduced compared to the case of t*_max_* = 5 s (Figure 3), while with a longer t*_max_* = 7 s, the distribution and level of collision point-risk is substantially increased.

The anisotropy is even more pronounced as a function of the bearing angle, *β*, of a pedestrian relative to the patient (Figure 4). If we assume that the patient is looking straight ahead most of the time, then angle *β* represents the prevalent eccentricity of the pedestrian on the patient's retina. Thus, the *β* angle at the peak of the risk function is the main parameter of interest in designing prismatic field expansion for PFL patients. It represents the optimal center of the residual island of vision to be provided by the prism.

In all cases presented so far, the risk density function peaks at *β* ≈ 45°. The risk density is very low for small bearing angles. Only those low-*β* pedestrians walking directly toward the patient may collide. This could be considered an underutilization of residual central vision, as even patients with very narrow residual central vision can see pedestrians at those small bearing angles, but most will not collide with the patient (under the reasonable constraints and simplifying assumption of infinitesimal width), but it does bode well for using part of the residual vision for prismatic field expansion, as described in Peli and Jung (2016).

The risk density increases with the increase in *β* toward the peak and declines past the peak, dropping substantially toward the higher bearing angles. Note that low risk at small bearing angles is true under the assumptions of equal probability of pedestrian walking directions, as may be the case in open space environments.

**Figure 6:**
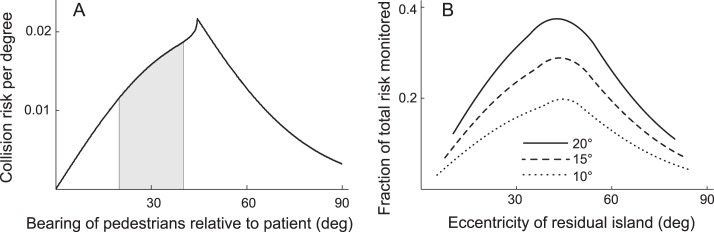
Fraction of risk monitored by islands of vision. (A) The collision risk density curve as a function of pedestrian bearing. The area under the curve represents the total collision risk posed by all pedestrians in any of the positions to the right of the patient (but not behind him). That is also the risk monitored by a normally sighted person, assuming a temporal field extent of 90°. The shaded area represents the fraction (31%) of that risk that would be monitored by a residual island of 20° centered at an eccentricity of 30° (achievable with a 57Δ prism). (B) The fraction of the total risk monitored by island windows of variable width as indicated, as a function of the island's center eccentricity (the shaded area of a sliding 20° window under the risk density curve of Panel [A], compared with corresponding curves for narrower windows [and the same risk density curve]).

The area under the risk density functions represents the total risk facing the patient from all possible pedestrians that meet the collision constraints. The risk density function shown in Figure 4 is reproduced in Figure 6A with the addition of a shaded section 20° wide centered at 30° eccentricity, representing the nominal width of a potential prism-created window of vision for a patient with ≈30° of residual central field (Peli & Jung, 2016). We say “nominal” because distortions in high-powered prisms both increase field of view via minification and limit it by total internal reflection, as described in Jung and Peli (2014) and taken into consideration in Peli and Jung (2016). The 30° window eccentricity illustrates the view achievable with the power of currently available prims that can be used for field expansion (57Δ, ≈ 30°). The ratio of the shaded area to the full area under the curve in Figure 6A is indicating that 31% of the total risk could be monitored by that island of vision. If prisms were available to create a 20° window centered at the peak of the risk curve (45°), 37% of the risk would be monitored. This represents 6% more of the absolute risk than can be monitored with currently available prisms but it is more than a 20% increase in effectivity of the higher power prisms. Figure 6B shows the fraction of the risk that can be monitored and thus mitigated by natural or artificial islands of 10, 15, and 20° width (corresponding to residual central fields of about 15°, 22.5°, and 30°, respectively, for prism-created islands), shifted to various eccentricities. Note that although the total risk varies substantially with the assumed t*_max_*, the fractional risk that can be monitored by an island of residual vision is independent of t*_max_*, because the risk density function is independent of t*_max_* (as shown in Appendix A). The fractional risk curves shown in Figure 6B are thus also independent of t*_max_*. Currently available Fresnel prisms provide a smaller than ideal but still substantial benefit.

**Figure 7:**
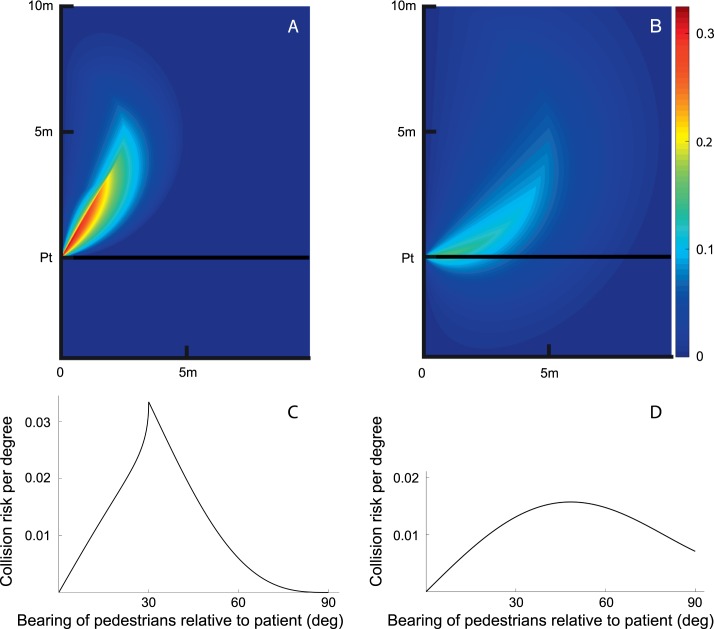
The effect of pedestrian speed range on the risk as a function of bearing angle from a patient walking at a speed of 1 m/s. (A) The risk function for slower pedestrians walking in the speed range of 0.5 to 1.0 m/s (or 0.5 to 1.0 times the speed of the patient). The risk in this case is shifted centrally and peaks at 30°. (B) The risk function as a function of eccentricity calculated for faster pedestrians walking at speeds between 1.0 and 2.0 m/s is flatter, peaking at farther eccentricity (49°) and extending to eccentricities behind the patient. (C) The risk density function for the conditions of (A). (D) The risk density function for the conditions of (B). Note that these density functions are independent of t*_max_*.

While the fractional risk monitored by an island is independent of t*_max_*, the risk density and the fractional risk monitored by an island of given width are sensitive to the pedestrian speed range. Risk from slower pedestrians is higher at lower bearing angles (more centrally), while faster pedestrians pose a higher risk when they are farther peripherally (Figure 7). In the limit, of course static pedestrians are a collision risk if they stand in the path of the patient. However, slow pedestrians will be detected at central eccentricities within the central residual island of vision and not by the peripheral islands. On the other hand, the risk from faster pedestrians is shifted peripherally. The risk from faster pedestrians extends to those that overtake the patient from behind. Since these results depend on the ratio of the pedestrian speed to patient speed, conclusions we draw regarding fast pedestrians apply equally to slow-walking patients, and vice versa.

Slow walking protects the patient by reducing the risk density from pedestrians ahead, while increasing the risk from pedestrians behind. Similarly, fast walking patients will face risk more centrally. (A patient we know said he walks quickly just so the most likely hazards will be within his central residual field.)

### Residual islands

Observation of the visual fields of the 42 patients with residual peripheral islands (shown in Appendix B) reveals a number of characteristics. We only used data from a single visit by each patient, so we have no direct information about the within-patient course of PFL. Nonetheless, the results from this sample of patients with RP and RP-like diagnoses largely seem consistent with a rather simple description of typical progression. The perimetric results of both eyes of most patients were quite symmetrical, indicating a similar progression of the midperipheral loss in both eyes. As the normal visual field extends farther temporally than nasally and farther down than up, the residual islands, which primarily start as a complete ring around the mid periphery ring scotoma, tend to lose the upper and nasal portions earlier, leaving the temporal and lower residual islands until further progression. Since the normal nasal field extends only to eccentricities of about 55° (Good, Fogt, Daum, & Mitchell, 2005), the residual nasal islands—when they exist—appear centered at an eccentricity of about 45°. The temporal residual islands are centered at about 75° eccentricity, as shown below. The asymmetry between the nasal and temporal residual islands results in complementary coverage of both eyes' lateral fields in binocular viewing, where the nasal residual field of the LE compensates substantially for the temporal mid periphery field loss of the RE and vice versa.

**Figure 8:**
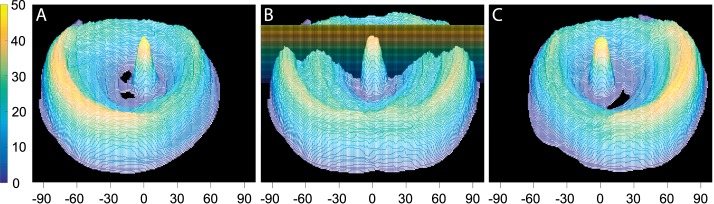
Histogram of field coverage of the 42 patients with residual islands. The height and the color coding mark the number of patients out of 42 that were seeing within each 1° × 1° cell. (A) The histogram for the LE. (C) The histogram for the RE. (B) The binocular histogram represents the union of the LE and RE panels. The horizontal axis represents the eccentricity (degrees) in the visual field along the horizontal meridian. The plane cutting through the binocular field marks a slice through the histogram used to calculate the visual field coverage along the horizontal meridian, corresponding to the lateral eccentricities that might be relevant to detecting a colliding pedestrian.

We quantified the visual field eccentricities visible to our population of patients by counting, for each 1° × 1° location, the number of patients whose digitized Goldmann field was marked as seeing in those locations. This three-dimensional histogram was first developed separately for the LEs and REs (Figure 8A, C). To analyze the binocular fields, a cell was counted for each patient that was seeing in that field location with either the LE or RE. This analysis produced the histogram of Figure 8B. This binocular population field illustrates the complementary nature of the nasal and temporal residual islands, resulting in wide coverage of the field despite significant midperiphery loss in each eye.

### Pedestrian collision risk for patients with residual peripheral islands

**Figure 9:**
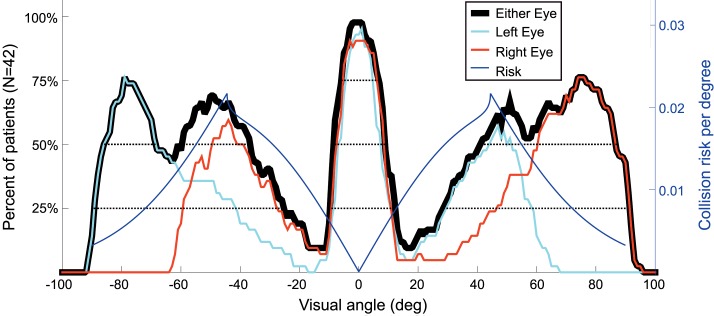
A slice through the data from Figure 8, showing the lateral angular extent of the residual islands with the calculated risk density function superimposed. The angular extents for the “either eye” data are marked at the 50% level, representing the minimum range of eccentricities monitored by half the patients. The peaks of the calculated risk density function (blue) closely match the locations of the nasal peaks of the patient peripheral islands data. Additional horizontal dotted lines mark the extent of the residual islands from both eyes at the lower and upper quartiles of the population.

Slices through the horizontal meridians for the LE, RE, and both eyes data of Figure 8 transferred to Figure 9 show the proportion of patients who had residual vision at various lateral eccentricities. Lateral vision (provided by natural residual islands or prism-created islands) can monitor for colliding pedestrians. The central residual island for this group is less than 20° in diameter, as that was a selection criterion. As can be seen, half the population had a residual nasal island at least 15° wide at about 45°, and about three quarters of the population had some temporal vision remaining at about 80°. The graph illustrates the wide coverage that the lateral residual islands provided in binocular viewing due to the asymmetry of the nasal and temporal islands, where for half the patients the eccentricities from below 40° and up to above 80° are visible on both sides. Superimposition of the collision risk density function (from Figure 6) in this figure illustrates that the naturally occurring residual islands can monitor and detect 75% of the collision risk for 50% of the patients in this sample. While the binocular slice in Figure 9 may suggest that some patients may have a double ring scotoma, such occurrence is rare and the second outer ring, when it exists, is very small in our population sample (see individual perimetry records in Appendix B).

As discussed above, very slow or static hazards are likely to collide more centrally. It is interesting to consider the role of the residual islands of our patient population in monitoring for such hazards. Static hazards will be monitored either by the residual central field (if they are as tall as the patient) or by the lower residual islands if they are at floor level (tripping hazards). The normal lower visual field extends to about 80° eccentricity (Good et al., 2005), and as a result the lower residual island, when it remains, appears centered at about 60°, extending for half of the patients between 38° and 72° in our sample population. This lower residual island enables a person of average height to monitor floor level hazards from about 0.6 to 1.6 m ahead, assuming primary gaze and little head scanning. This range is well suited for proper response. It is, however, difficult to achieve coverage of that high level of lower field eccentricity with prisms, as current prisms provide only half the power required, and the needed field coverage along the path is larger than the portion of the lower central residual field that might be used to monitor the lower field. As for the fast pedestrians coming from the side, we have no prism solution suitable for replacing the temporal field loss.

## Discussion

Simplifying assumptions in our risk model limit the accuracy of its predictions, but we nonetheless believe the general results will stand and they are informative. The speed range we have selected is reasonable, as are the limits to time to collision of interest. Slower pedestrians than modeled pose little threat, while faster pedestrians than modeled bear a social responsibility for avoiding collisions. Pedestrians who would not collide within the maximum time to collision assumed are simply not yet a concern (and changing the time to collision limit does not affect the risk density by bearing angle relationship that we need for our prism designs). The assumption that pedestrians walk in all directions with equal probability is only appropriate for the open space environment we considered, such as malls and parks. More constrained environments, such as hallways and narrow sidewalks, may be more common than the open environment we modeled, but the limited scanning that we know PFL patients do exhibit (Luo et al., 2008; Vargas-Martin & Peli, 2006) is fairly well suited for restricted environments. Hazards ahead are generally detected by residual central vision, and intersections cue wider head scans to detect crossing traffic. Normal scanning is not sufficient to deal with open environments, so optimizing prism aids per our model seems appropriate. Our treatment of patient and pedestrians as points underestimates the risk of collision, and a more complex model could take size into account. However, we have also assumed no scanning, while normal saccades may compensate for the difference between points and more realistic collision envelopes. Finally, our assumption of constant speeds and heading by the participants may seem unrealistic, but it is hard to imagine how to remove that constraint. However, at any given moment, any such course changes simply result in the identical diagram at an instant later in time.

Interestingly, the residual islands in our sample group of patients are an excellent match to the risk distributions found by our model (making testing of these islands potentially more predictive of results with prism-created islands). The nasal residual fields cover the area around the 45° eccentricity, while the residual temporal crescents cover the high eccentricities. The pericentral area is least covered by the residual islands. In our review of prior literature on the residual islands in RP we did not find any paper that illustrated or discussed the binocular combination of the residual islands. As we have shown, the binocular presentation reveals that the barely overlapping and highly complementary coverage of the nasal residual islands from one eye and the temporal residual islands from the other provides almost optimal coverage of the peripheral field, resulting in loss only in the mid-near periphery. Field loss in RP tends to start at the eccentricity where rod density is highest, so metabolic explanations of progress may account for the loss patterns. However, we do note that the effects of PFL are often not noticed until residual fields shrink below 40°diameter (a common observation about PFL, but not specific to RP). The complementary residual islands may forestall noticing the PFL even after the central field has shrunk to less than 40° (as it has in our sample), but we know of no data to substantiate that. Although the far periphery, representing the temporal crescents, has some overlap with the collision risk function, it is mostly limited to collisions with very close or very fast pedestrians, or other hazards like bicyclists, approaching from a bearing of 80° to 90°.

The risk from oncoming pedestrians approaching at low-bearing angles is the lowest. This suggests that a limited increase in central residual vision (as may be obtained by low-level minification) may only have limited benefit in avoiding pedestrian collisions. Alternatively, it can be stated that continuous shrinking of the central field has little impact on that risk, though clearly that does affect other functions. In relation to prism spectacles, this suggests that low-power prisms that will create residual islands abutting the central residual island (Woods et al., 2010) will not be of much use in avoiding pedestrian collisions.

The analysis of the risk for collision of patients with pedestrians revealed a highly nonisotropic risk as a function of bearing angle from patient path to pedestrian. There are several notable characteristics of this distribution. The highest risk is associated with a lateral retinal eccentricity of about 45°. This suggests that our prism devices will be most effective if they can create artificial residual islands at that eccentricity. Currently, the prisms that we have commercially are limited to 30°. While that reach is suboptimal, it does cover a fairly high risk area in the distribution and thus may have a meaningful benefit. We have already designed and made prototypes of a few configurations of high-power prism devices that may reach closer to the 45° peak of the risk distribution (Peli, Bowers, Keeney, & Jung, 2016).

The perimetry analysis tools we developed may be useful in answering other research questions using the large patient research database we tapped for our small sample. For instance, we noted that the initial ring scotoma seems to be centered on the blind spot, not the fovea, but as it grows, the inner boundary migrates toward the fovea. Thus the central residual field usually becomes circular and centered on the fovea. The outer boundary expands to the left and right in roughly equal measures from the optic nerve head, so that the lateral peripheral residual islands are symmetric around the blind spot. We had not seen that reported in the literature, and perhaps that provides clues to the nature of the underlying loss mechanisms and possibly for retinal development. This does account for the asymmetry of the nasal and temporal islands. With the blind spot about 15° temporally, temporal and nasal residual fields that are equidistant from it should be about 30° different in eccentricity from the fovea, as indeed we found them to be (about 75° and 45°, respectively).

While studying residual islands may seem unnecessary for our primary interest in understanding where best to target artificial islands of vision (per our model), we undertook the investigation to first understand whether narrow natural peripheral islands might be effective in detecting pedestrian hazards, for if they are not, there would be little hope for the (likely smaller) artificially created islands. Note, however, that the prism-created artificial islands are imaged on a much more central and thus higher sensitivity retinal area. We plan to first test patients with natural islands without prisms before testing prism-created artificial islands for PFL subjects without the peripheral residual islands.

Even though our investigation was based on a modest sampling, it provided consistent quantification of the shape, location, and size of these features, filling a significant gap in the literature and raising questions that may provide insights into the natural development of the visual system.

The value of our observation regarding the optic-disk-centric development and possibly susceptibility of the visual system to the loss in RP may be appreciated by examining prior reports about the topography of ganglion cells (Curcio & Allen, 1990) and the neural bandwidth and veridical perception across the retina (Wilkinson, Anderson, Bradley, & Thibos, 2016). Both papers reported a nasal–temporal asymmetry in their measurements. In both, the nasal retina showed higher cell density and higher sensitivity than the temporal retina. Despite noting the asymmetry and even plotting the optic nerve head within the nasal retina in a few of the figures, neither paper commented in any way about a possible relationship between the position of the optic nerve head and the asymmetries found. Wilkinson at al. also noted a small vertical asymmetry in their highly accurate data, where the superior retina exhibited slightly higher sensitivity than the lower retina. That is an effect that is consistent with the slightly higher position of the optic nerve head on the retina. A similar effect was noted in Curcio and Allen's figure 6B and D. The higher density of ganglion cells in the superior retina is even more pronounced in the more nasal retina above the optic nerve head (their figure 5A). Wilkinson et al. missed that relationship, possibly because they incorrectly plotted the optic nerve head position (obtained from Goldmann perimetry) in its lower field-coordinates position rather than the higher retinal-coordinates position. (The authors confirmed that it was a mistake; Larry Thibos, personal communication, 2016.) It is impressive that both papers had data of such high accuracy that it reflected the mere 2° vertical shift in the position of the optic nerve head. The visual streak-like characteristics identified for the nasal retina in both papers provide increased sensitivity in the far temporal field, possibly evolved to detect fast moving predators, as suggested by our model. At the same time, limiting the higher sensitivity of the nasal field to about 45° provides additional binocular overlapping coverage for the peak of the collision risk with other pedestrians moving at the same speed. In all, this also provides an additional explanation for the position of the optic nerve head, as an alternative to the explanation offered by Arditi (1987).

Our collision risk model is an interesting and valuable geometric analysis of pedestrian collision threats under reasonable assumptions, but it is independent of, and cannot say anything about, how effectively this information can be used. This analysis may have applications beyond our current path and may spark the development of additional models. The benefits for rehabilitation of patients with PFL will depend on the effectiveness of the prism glasses we are developing based on the model. We know that visual field expansion via our peripheral prism glasses for hemianopia has been successful (Bowers, Keeney, & Peli, 2008, 2014; O'Neill et al., 2011), and our Peli and Jung (2016) paper identifies paths and reasons to believe that success can be achieved for expanding the visual fields prismatically for other situations, including severe PFL, meeting the challenges identified in Apfelbaum and Peli (2015). Finally, in Peli, Bowers, Keeney, and Jung (2016) we have identified a few options for developing higher power prisms that should provide more effective solutions to the mobility problem we have addressed here.

## Supplementary Material



Supplement 1
