# Supplementary figures and images for: The risk of pedestrian collisions with peripheral visual field loss
Source: J Vis. 2016 Dec 5;16(15):5. doi: 10.1167/16.15.5 (PMC5142795; doi:10.1167/16.15.5)

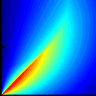

Supplement: Supplementary file 1 [file jovi-16-14-26_ICON.gif]
