# Supplementary material for: The risk of pedestrian collisions with peripheral visual field loss
Source: J Vis. 2016 Dec 5;16(15):5. doi: 10.1167/16.15.5 (PMC5142795; doi:10.1167/16.15.5)
Supplement: Supplement 1 [file jovi-16-14-26_s01.pdf]

Appendix A: Calculating and Diagramming Point Risk

Note: Figures A1 and A2A here are similar to the first two figures in the main text. They are replicated here for the convenience of the reader.

Notations (Fig. A1):

P, as the Patient

D, as a peDestrian, and (D_x, D_y) indicates its coordinates

C, as Collision point

The patient walks straight from P_0 to C. The pedestrian walks straight from D_0 toward the patient's path at various possible angles, α_i, toward various corresponding points of collision, C_i.

For each α_i, walking at a constant speed, r_i, required to collide at C_i:

α is the angle at the pedestrian from P_0 to the collision point (angle P-D-C)

β is the bearing angle from the patient to the pedestrian (angle C-P-D)

Note however, that since P and D both walk at constant speeds and headings during a walk to collision, $\alpha = \alpha_0$ and $\beta = \beta_0$ throughout the walk, as the P-C-D triangles remain similar as their area and the distances shrink.

d, as distance

t, as time (usually the time to collision, with t_{max} as an imposed constraint)

r, as rate (walking speed)

s, (slow), the low speed constraint; D cannot walk slower than r_s

f, (fast), the high speed constraint; D cannot walk faster than r_f

i, a subscript variable representing a particular collision point or its corresponding α heading angle. (i = 1, 2, 3)

A collision will occur if the pedestrian, D, and the patient, P, arrive at C at the same time. The imposed constraints on realistic speeds limit the pedestrian headings (angles α) for which collisions can occur. In this appendix we derive these constrained angles and their corresponding collision points C on the patient's path, given D_0, β, and pedestrian speed range of interest.

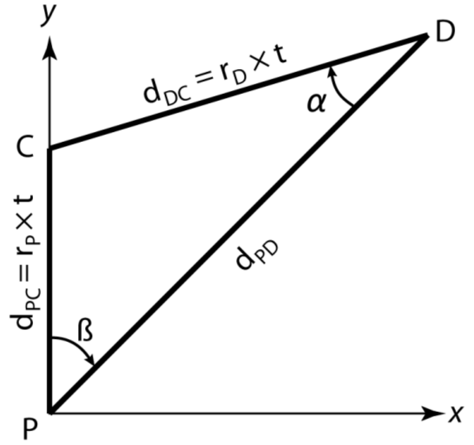

Figure A1: The geometry of a collision. Patient, P, is walking straight forward toward the collision point, C. PeDestrain, D, at a bearing angle, β, is walking towards the same collision point. His heading is marked by angle α relative to P. As both are progressing their positions maintain similar triangles, keeping all angles fixed and only changing the distances.

Constraints

$0° < \beta < 180°$. Results for the other half-plane (left of the patient) are symmetric.

$-\beta < \alpha < (180° - \beta)$. D must head toward the P path.

While the model shows the speeds P must take to collide with D at all point on D's path, only headings for which $r_s \le r_D \le r_f$. are considered at risk. Colliding with D at speeds outside that range is not allowed, and the full wedge diagram (Figure A2A) indicates which constraint would be violated for every disallowed pedestrian heading. We also require $r_s \le r_P \le r_f$ to ensure that the case $r_{P} = r_D$ is included as a risk (time to collision permitting).

t_{max} is the longest time to collision that we consider in our analyses and examples.

Solving the P-C-D Collision Triangles

By the Law of Sines,

$$\alpha = \sin^{-1}(\sin(\beta) \cdot r_P / r_D) \quad \text{(A1)}$$

where r_P / r_D is the key simplification of d_{PC} / d_{DC} that results because $t_{PC} = t_{DC}$.

Also by the Law of Sines,

$$d_{PC} = d_{PD} \cdot \sin(\alpha) / \sin(180 - \beta - \alpha). \quad \text{(A2)}$$

Since P starts at (0,0),

$$t_{DC} = t_{PC} = d_{PC} / r_{P}, \quad \text{(A3)}$$

and

$$d_{DC} = d_{PC} \cdot r_D / r_P. \quad \text{(A4)}$$

Observations

Solving Equation A1 for the ratio r_D / r_P that will result in a collision gives:

$$r_D / r_P = \sin(\beta) / \sin(\alpha).$$

For any given β and r_P, the slowest r_D that will end in a collision is at $\alpha = 90°$, and

$$r_{min} = \sin(\beta) \cdot r_P. \quad \text{(A5)}$$

At a slower speed the pedestrian will arrive at the path after the patient has already passed there.

Forming the Wedge Diagram (Calculating the limiting edges of the wedges)

If $r_f < r_{min}$, D is too far from the P path to collide at any speed in the constraint range we are analyzing. There is no wedge diagram in that case.

The pedestrian will never collide with the patient if $\alpha < 0$, as that is directed behind the patient's starting point. That region is labelled "BELOW START

C_1:

Limiting D's maximum speed sets the closest point to P_0 they can collide. A collision at C_1 is reachable when D walks at the highest allowable speed, r_f. For the collision to occur, $d_{PC1} / r_P = d_{DC1} / r_f$.

Thus, by Equation A1, the heading relative to P and the bearing line D takes, α_1, is given by

$$\alpha_1 = \sin^{-1}(\sin(\beta) \cdot r_P / r_f), \quad \text{(A6)}$$

the wedge between the bearing line and α_1 is labelled "D CAN'T GO THIS FAST".

C_2 and C_3:

If D starts sufficiently close to the P path, the speed D must walk to collide would have to be less than the slowest speed permitted, r_s, and a gap exists along the P path where collisions with these slow D walks are excluded. There are two solutions to the arcsine in Equations. A1 and A6 for $r_D = r_s$, yielding α_2 and α_3. If $r_{min} > r_s$, there is no gap, as it is not necessary for D to walk that slowly to collide anywhere along the P path. As a corollary, when $\beta > 90°$ (D overtaking P from behind) there can be no gap, since D must walk farther and hence faster than P to collide, r_D must be greater than r_P, and thus greater than r_s ($r_s \leq r_P$).

The low-speed gap, if present, is symmetric about α_{min} (= 90°) relative to the bearing line, since the two values of arcsine (in quadrants1 & 2) are symmetric about 90°.

The wedge between α_1 and α_2 is labelled “COLLIDING”, and the wedge between α_2 and α_3 is labelled “D CAN’T GO THIS SLOW”.

C_{max}:

C_{max} is the highest collision point, reached when P has travelled for the maximum time to collision constraint, t_{max}. Thus C_{max} sets an upper bound on all collision points. Although the example in Figure A2A illustrates C_{max} above C_3, for farther pedestrians C_{max} decreases and can fall below any C_i. So

$$C_{max} = r_P \bullet t_{max}, \qquad \text{(A8)}$$

and after using the Law of Cosines to calculate d_{DCmax}

$$\alpha_{max} = \sin^{-1}(\sin(\beta) \bullet C_{max} / d_{DCmax}). \qquad \text{(A9)}$$

If $\beta > 90°$ or $C_{max} < C_{rmin}$, the arcsine solution in the first quadrant applies. Otherwise, it is the solution in the second quadrant that is needed.

The wedge between α_3 and α_{max} is labelled “COLLIDING”, and the wedge from α_{max} to $\alpha = 180° - \beta$ is labelled “TIME LIMITED”.

Point Risk

Point risk is calculated as:

$$((\alpha_2 - \alpha_1) + (\alpha_{max} - \alpha_3)) / 180°. \qquad \text{(A10)}$$

Any $\alpha_i > \alpha_{max}$ should be replaced by α_{max} for this calculation. If there is no low speed gap, the equation becomes:

$$(\alpha_{max} - \alpha_1) / 180°. \qquad \text{(A11)}$$

Effect of t_{max} on point risk, bearing risk and risk density

Although t_{max} clearly affects point risk (Eq. A10), we now show that all bearing risks scale identically with t_{max}, and collision risk density is thus unaffected by t_{max}. When t_{max} is scaled by a factor k, the point risk at a distance $k \bullet d_{DC}$ along a given bearing angle is identical to the point risk at unscaled distance d_{DC}. Using the same notation as above, we prefix k to subscripts to indicate that they refer to the value at k times the unscaled d_{DC} when t_{max} is scaled by k. Thus, for a given β and unscaled d_{DC}, the t_{max}-limiting α_{kmax} angle is (from Eq. A9) given by:

$$\alpha_{kmax} = \sin^{-1}(\sin(\beta) \bullet C_{kmax} / (k \bullet d_{DC})). \qquad \text{(A12)}$$

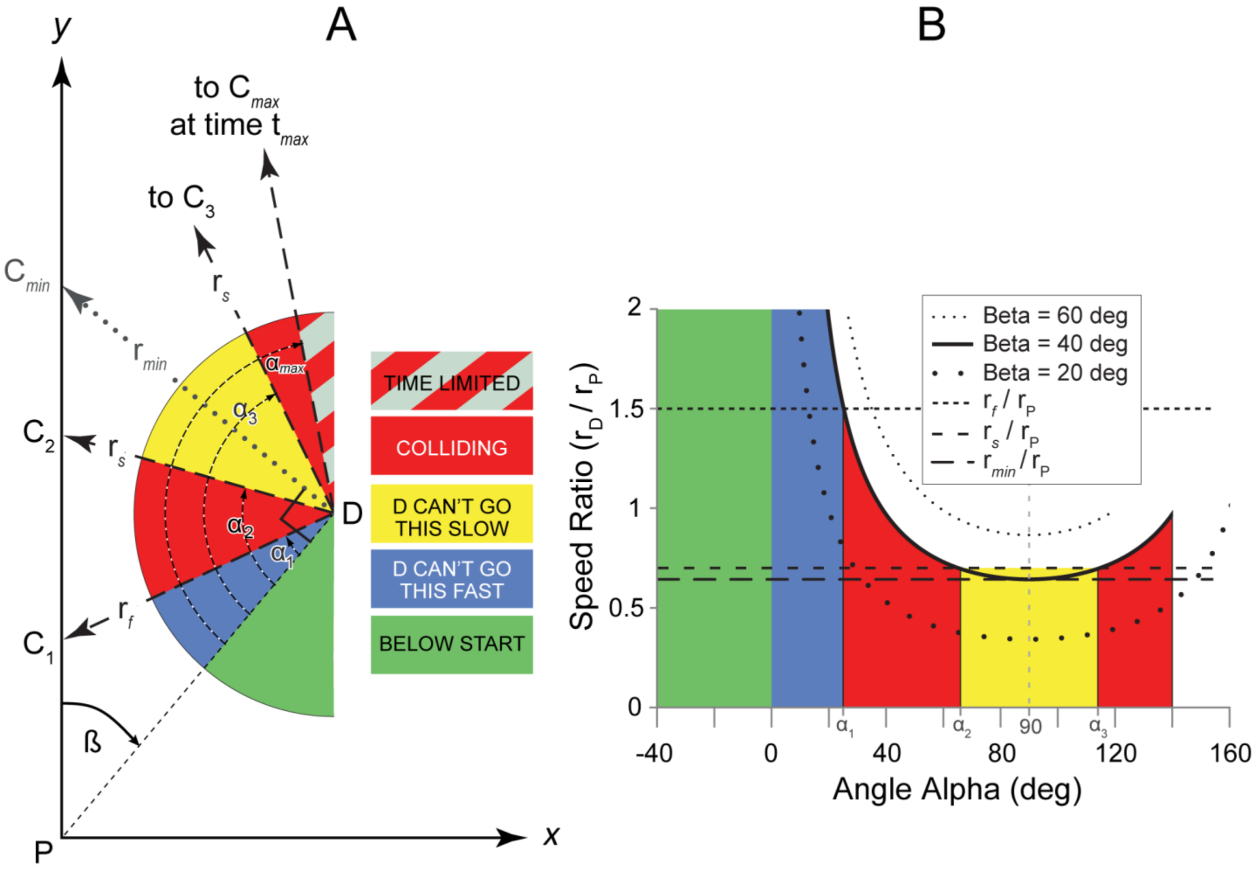

Figure A2. (A) The Wedge Diagram. The angles illustrated are for β = 40°, d_{PD} = 1.3m, r_P = 1m/s, r_s = 0.7m/s, r_f = 1.5m/s, and t_{max} = 5s. Except for the effect of the t_{max} time limit (as seen in the gray-striped area), the diagram would be identical for any other d_{PD} along that bearing angle, and for any other equal values of r_s/r_D or r_f/ r_D, as follows from the equations below. (B) How Pedestrian speed varies with heading angle. The shaded ranges correspond to the wedges of the same color in Figure A2A for the β = 40° curve. Heading at α = 0°, with respect to the bearing line from P to D, D would have to travel at infinite speed to collide with P (at the starting point). The collision curves do not exist for α < 0°, as D would be headed behind the P starting point. As α increases and D heads farther up the P path, P's speed must decrease in order to collide. The first collision occurs when the speed decreases to r_f at α_1. Collisions are possible as D slows further to r_s at α_2. Between α_2 and α_3, where the curve falls below r_s, collisions do not occur because D would have to go slower than the low speed limit. That is the "low speed gap." If β is large enough, there is no low speed gap, as r_{min}, the slowest speed D can go and still meet up with P, is above r_s. As α increases past the minimum speed at α_{min} = 90°, collisions can again occur. Speed then increases, until, at α = 180° - β. The pedestrian is heading parallel to the P path, walking at the same speed as P to collide at infinity. Note that the curves are independent of d_{PD}, as they are the same all along a bearing. If, on the other hand, we fix a value for r_P, as was done in (A), it would be possible to illustrate the effect of t_{max}. For the example parameters in (A), the gray striping for the t_{max}-limited region would extend in (B) from α = 128° to α = 140°.

Since P travels k times as far to reach the limiting collision point, $C_{kmax} = k \cdot C_{max}$, and

$$C_{kmax} / (k \cdot d_{DC}) = C_{max} / d_{DC}) \qquad (A13)$$

Therefore,

$$\alpha_{kmax} = \alpha_{max}. \qquad (A14)$$

Since all other angles in the wedge diagram remain unchanged by distance along a bearing, each $\alpha_{ki} = \alpha_i$ (for i = 1, 2, 3) and thus the point risk value at $k \cdot d_{DC}$ is the same as the point risk value at d_{DC}, when $t_{max.}$ is scaled by k.

Thus, for any given bearing β, a plot of point risk as a function of d_{DC} would look identical as a plot for point risk as a function of d_{DC} when t_{max} is scaled by k, except that the X axis would be scaled by k and it would extend k times as far before reaching the value where point risk becomes zero (when α_{kmax} becomes less than α_1).

Alternatively, the point risk value at d_{DC}, when t_{max} is scaled by k, is the same as the unscaled point risk value at d_{DC}/k. The range of point risks remains identical.

While the range of point risk values along a bearing is unchanged when t_{max} is scaling by k, the bearing risk slice extends k times as far and the area of the slice is scaled by k_2. The bearing risk for the scaled slice is thus scaled by k_2. The bearing risk calculation for all bearing slices scales in the same way, and, when each is divided by the sum of the bearing risks, yields an identical risk density function. Collision risk density is thus independent of t_{max}.

Appendix B: Goldmann Fields

Goldmann fields for all patients, including diagnosis.
In a few cases, in one eye, the peripheral island was partially connected to the central island. This had negligible effect on our analyses presented here and therefore these cases were included.

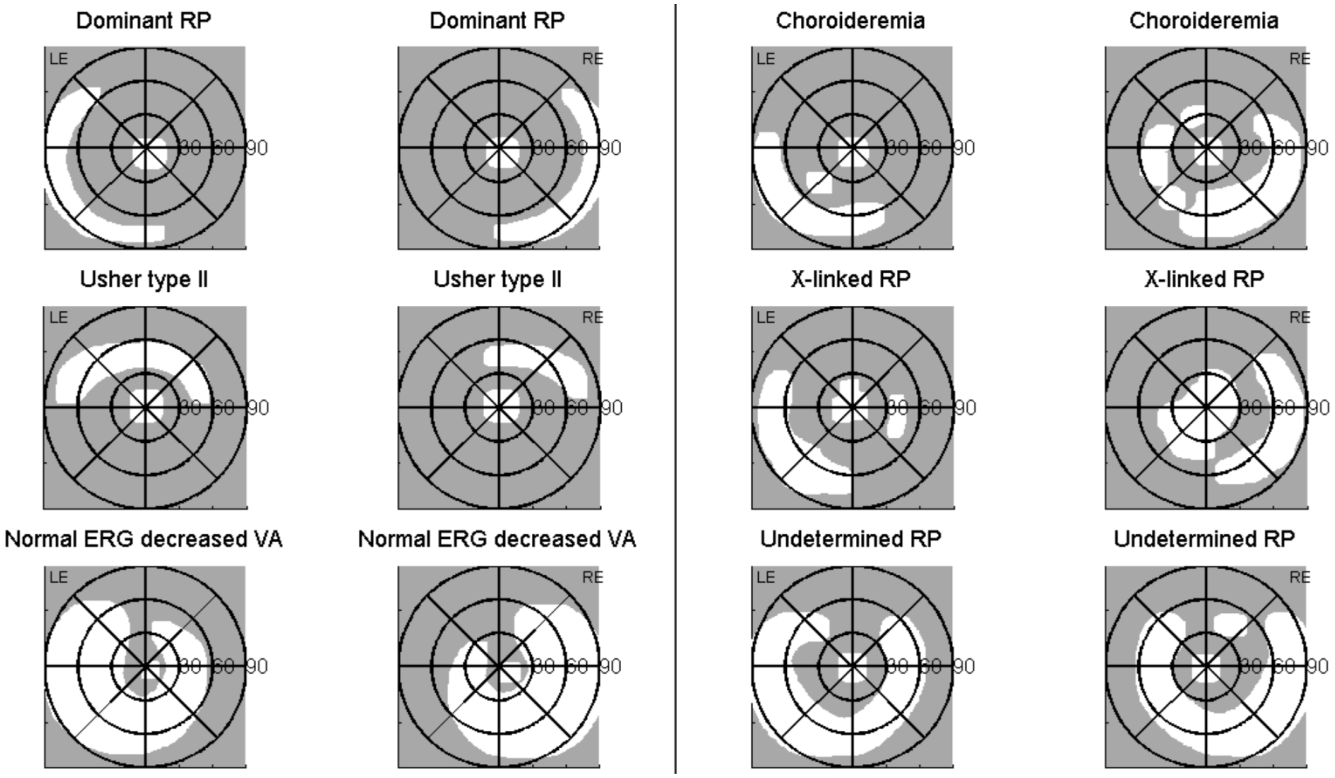

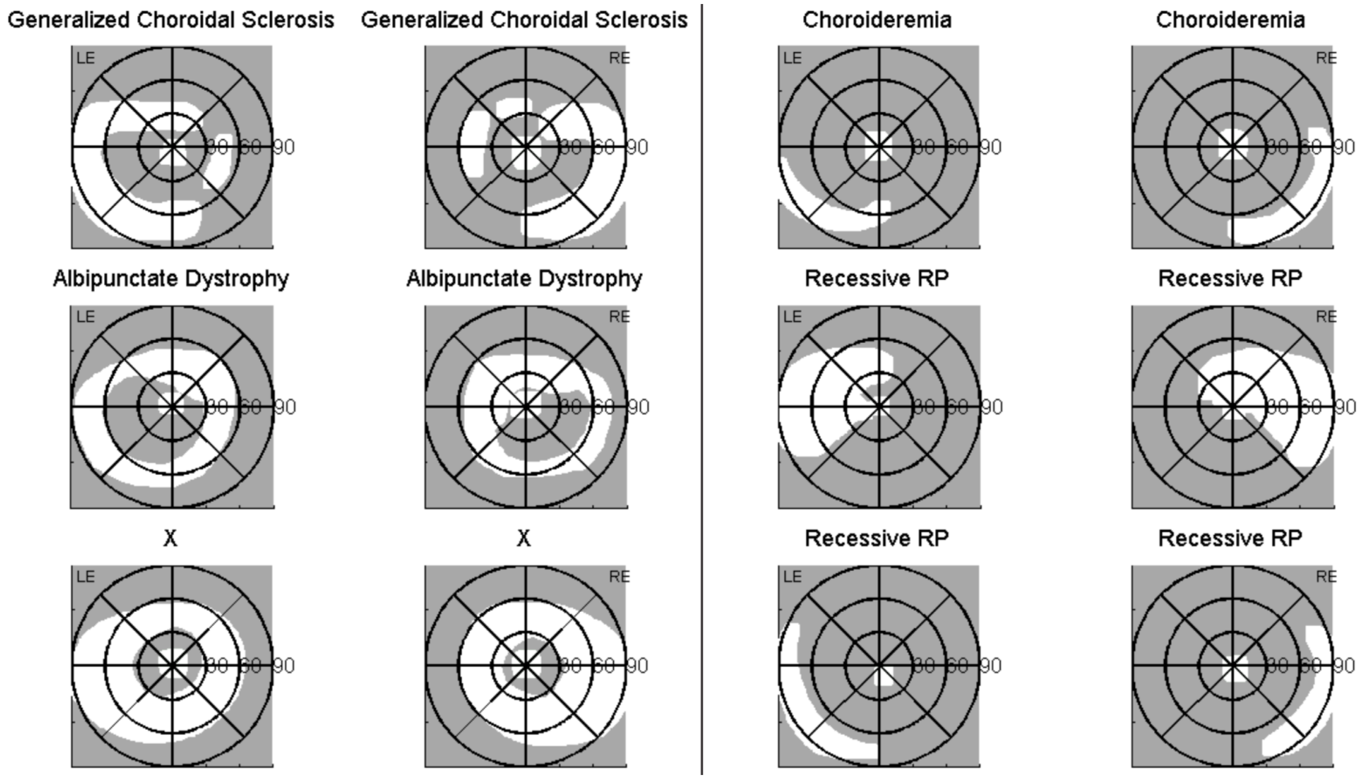
Generalized Choroidal Sclerosis
Generalized Choroidal Sclerosis
Choroideremia
Choroideremia
Albipunctate Dystrophy
Albipunctate Dystrophy
Recessive RP
Recessive RP
X
X
Recessive RP
Recessive RP
LE
RE
30
60
90

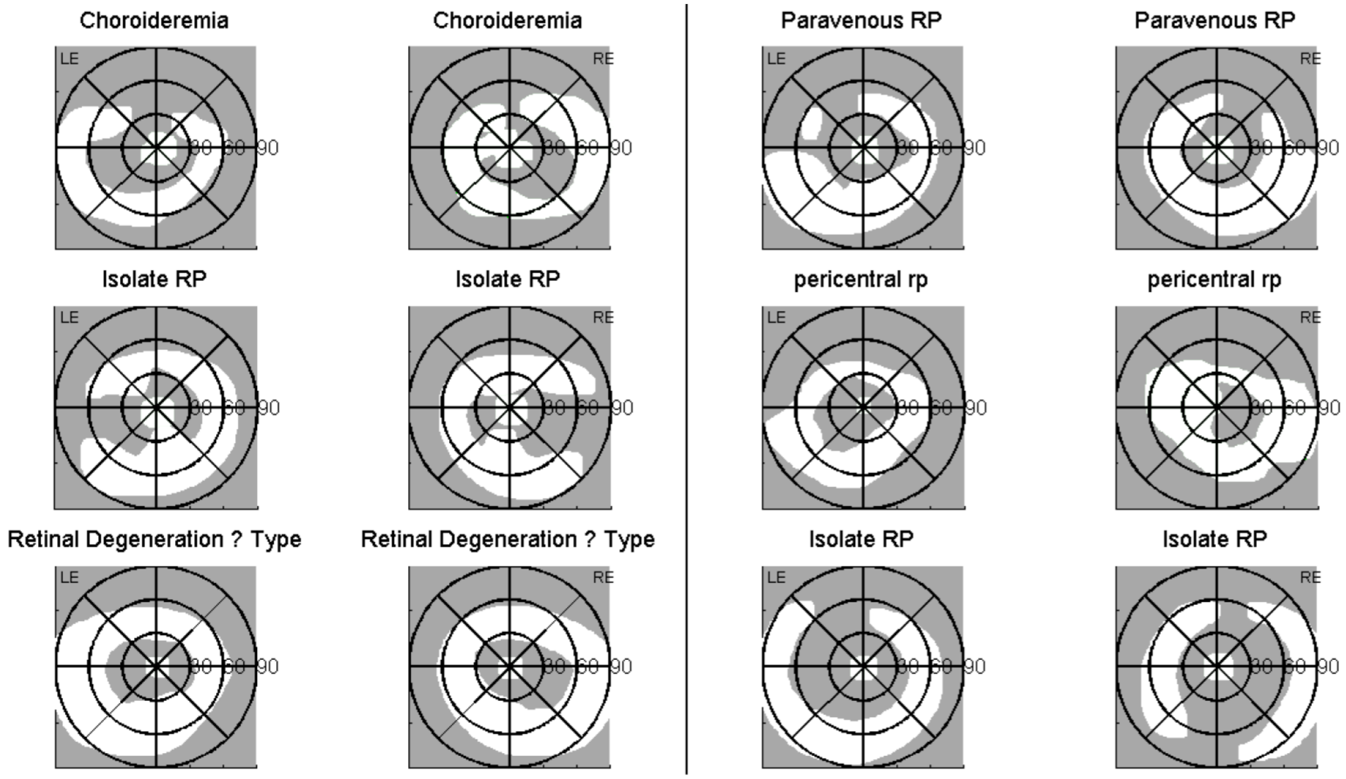
Choroideremia
Choroideremia
Paravenous RP
Paravenous RP
Isolate RP
Isolate RP
pericentral rp
pericentral rp
Retinal Degeneration ? Type
Retinal Degeneration ? Type
Isolate RP
Isolate RP
LE
RE
30
60
90

Recessive RP

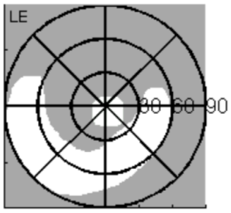

Recessive RP

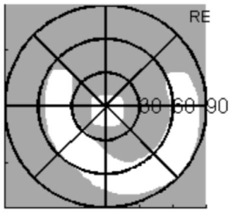

Pericentral RP

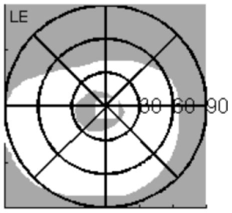

Pericentral RP

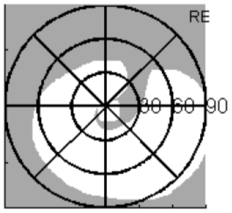

Usher Syndrome type II

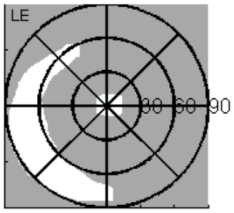

Usher Syndrome type II

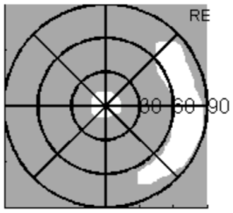

Cone Rod Dystrophy

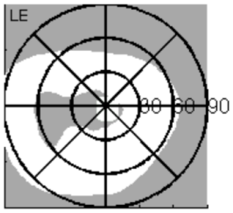

Cone Rod Dystrophy

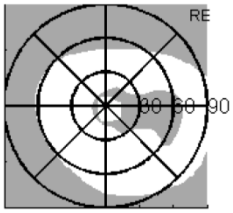

Pericentral RP

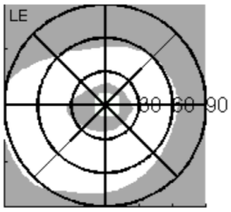

Pericentral RP

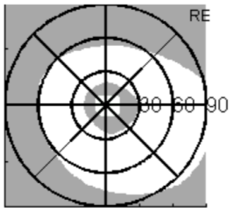

Cone Rod Dystrophy

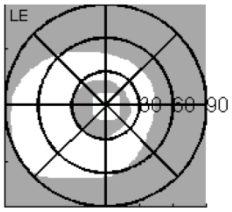

Cone Rod Dystrophy

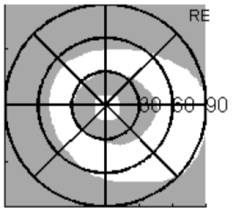

Isolate RP

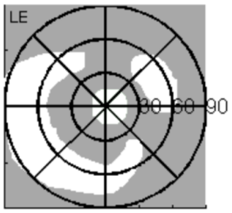

Isolate RP

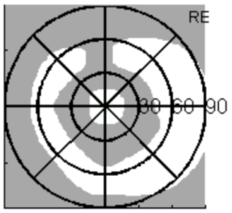

Usher type II

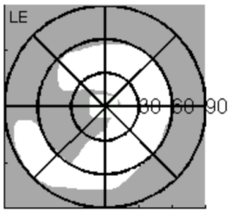

Usher type II

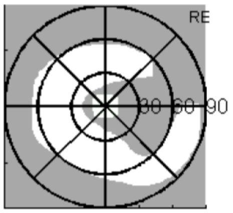

Dominant RP

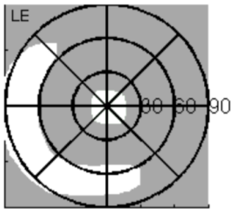

Dominant RP

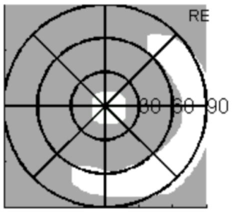

Undetermined RP

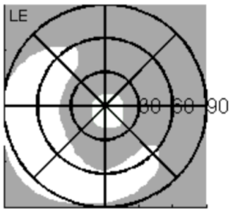

Undetermined RP

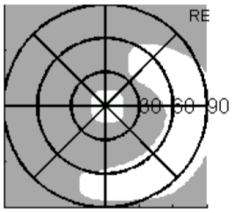

X

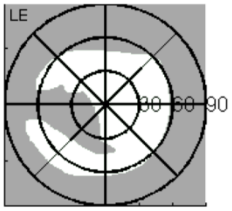

X

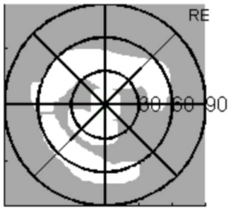

Dominant RP

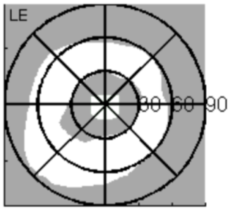

Dominant RP

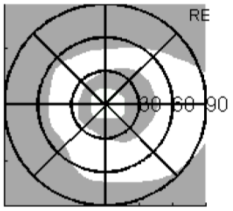

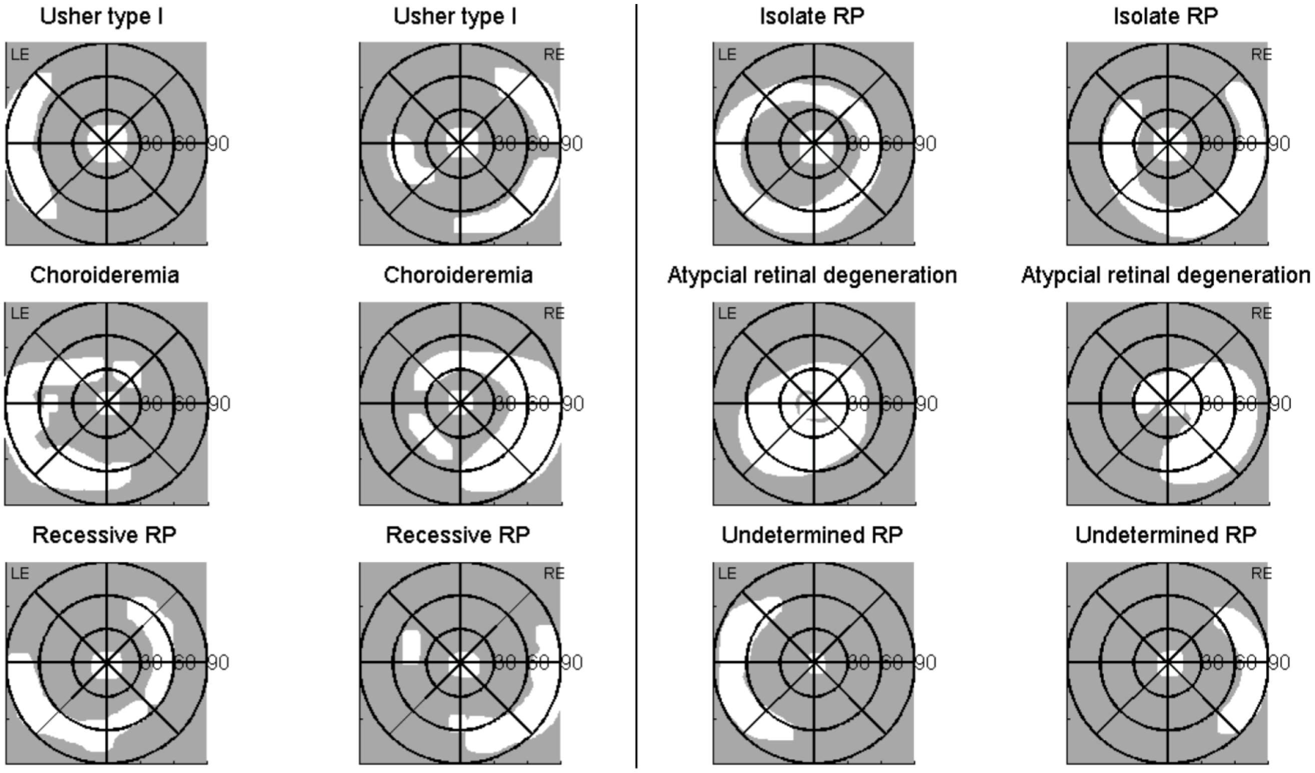
Usher type I
Usher type I
Isolate RP
Isolate RP
Choroideremia
Choroideremia
Atypcial retinal degeneration
Atypcial retinal degeneration
Recessive RP
Recessive RP
Undetermined RP
Undetermined RP
LE
RE
30 60 90

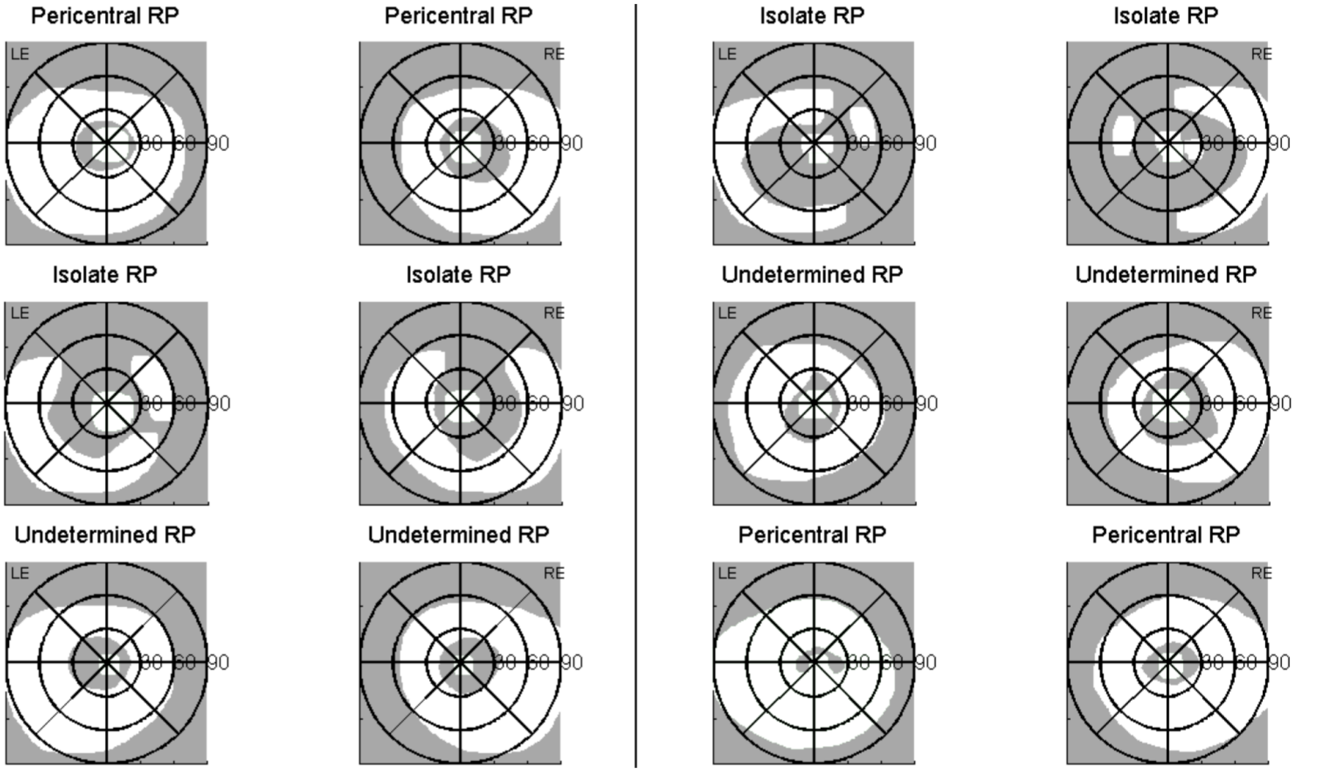
Pericentral RP
Pericentral RP
Isolate RP
Isolate RP
Isolate RP
Isolate RP
Undetermined RP
Undetermined RP
Undetermined RP
Undetermined RP
Pericentral RP
Pericentral RP
LE
RE
30 60 90
